# Dietary fatty acids and gallstone risk: insights from NHANES and Mendelian randomization analysis

**DOI:** 10.3389/fnut.2024.1454648

**Published:** 2024-08-15

**Authors:** Minghe Wang, Jintao Guo, Siyu Sun

**Affiliations:** Department of Gastroenterology, Shengjing Hospital of China Medical University, Shenyang, China

**Keywords:** fatty acid, gallstone, cross-sectional study, NHANES, Mendelian randomization

## Abstract

**Background:**

Prior research suggests polyunsaturated fatty acids (PUFA) may prevent gallstones, but evidence on saturated fatty acids (SFA) and monounsaturated fatty acids (MUFA) is limited. This study aims to explore the associations between fatty acids and gallstones using a large sample of American population and Mendelian randomization (MR) methods.

**Methods:**

The cross-sectional study involved 6,629 participants from the National Health and Nutrition Examination Survey (NHANES) 2017–2020. Logistic regression and restricted cubic spline (RCS) analysis were conducted after stratifying by gender subgroups. Two-sample MR analysis was used to explore the causal relationship between fatty acids and gallstones without confounding factors.

**Results:**

In females, higher SFA intake was positively associated with gallstone risk, while higher intake of n-3 and n-6 PUFA was negatively associated. No significant associations were found in males. No nonlinear correlations were found in any group by RCS analysis. MR analysis indicated that SFA, n-3, and n-6 PUFA could reduce gallstone risk.

**Conclusion:**

The influence of dietary fatty acid composition on gallstone development differs by gender, providing insights into dietary prevention and treatment of gallstones.

## 1 Introduction

Gallstones are a globally prevalent digestive disease, affecting 10–20% of individuals in developed countries and often presenting without obvious symptoms in the early stages (1). However, as the disease progresses, serious and potentially life-threatening complications may occur, including acute cholangitis, cholecystitis, and biliary pancreatitis (2–4). The high incidence and complications of gallstones impose significant economic and health burdens, necessitating effective preventive and curative measures.

Gallstone formation is influenced by genetic predisposition, lipid metabolic disorders, and lifestyle habits, with genetic factors contributing only 25% to the risk, emphasizing the importance of environmental and lifestyle influences (1, 5, 6). Growing emphasis is placed on the influence of dietary fatty acids on gallstone formation. Prior research has explored the potential protective role of polyunsaturated fatty acids (PUFA) in preventing gallstones (7–10). However, the evidence on saturated fatty acids (SFA) and monounsaturated fatty acids (MUFA) is limited by small sample sizes and the influence of confounding factors.

This study conducted a comprehensive investigation using publicly available databases. The relationship between dietary fatty acids and gallstones was explored in a large population sample using National Health and Nutrition Examination Survey (NHANES) data. Mendelian randomization (MR) was then applied to investigate the causality of fatty acids on gallstones, adjusting for residual confounding factors and addressing the limitations of the cross-sectional study. This study aims to provide scientific evidence for preventing gallstones through dietary intervention.

## 2 Methods

### 2.1 Cross-sectional study

#### 2.1.1 Study population

NHANES is a continuous research initiative by the National Center for Health Statistics (NCHS), gathering data on characteristics, dietary habits, and health conditions of American participants. The study obtained ethical approval from the NCHS review committee. The NHANES data are collected using multi-stage stratified sampling, ensuring comprehensive national representation.

**Figure 1:**
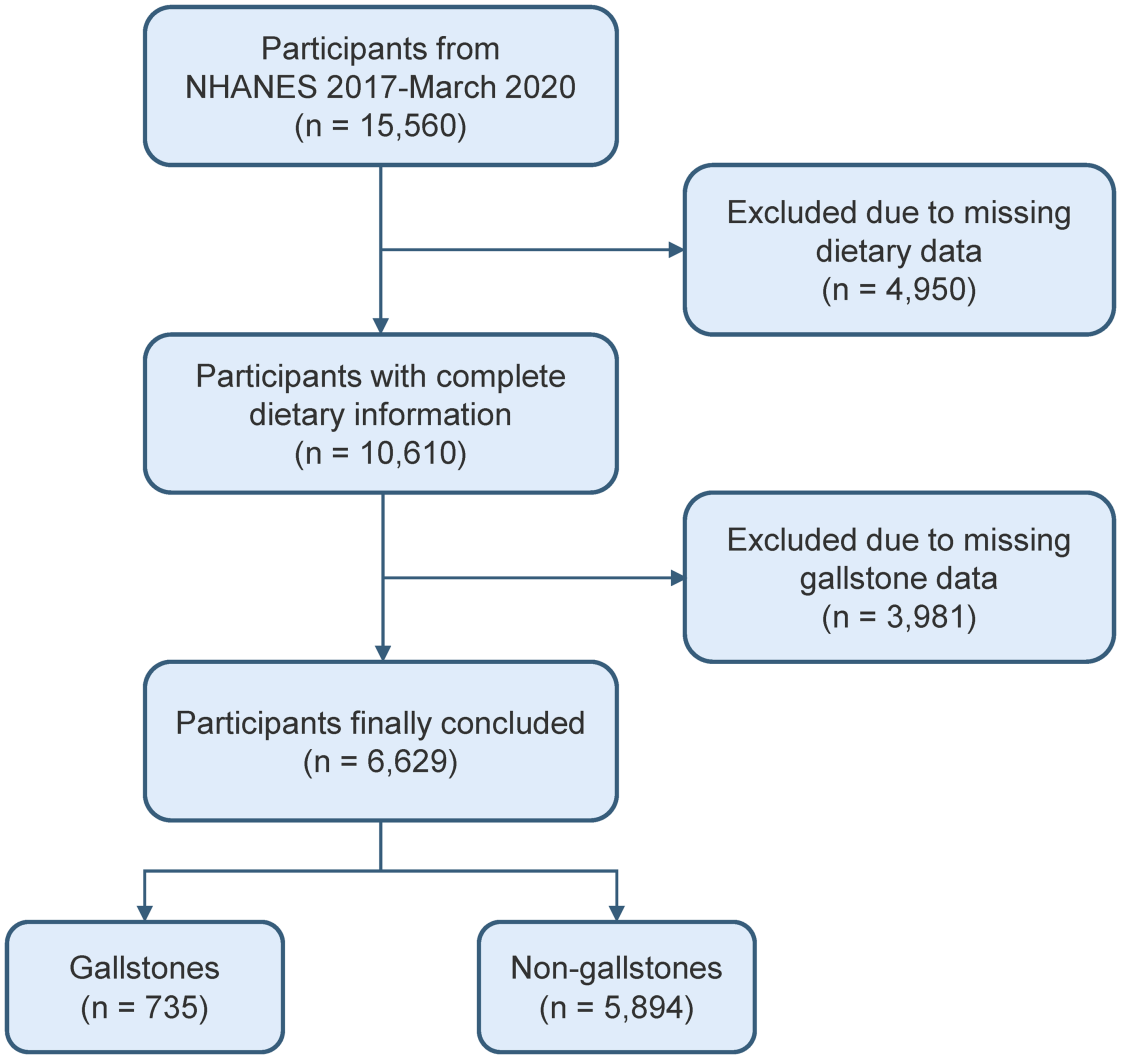
Flowchart of participant selection.

This study involved a cohort of 15,560 people from the 2017–2020 cycle, as the gallstones questionnaire was only available during that time period. Since the gallstones questionnaire was only administered to individuals over 20 years old, those under 20 were excluded from this study. After excluding missing dietary data and gallstone information, 6,629 participants were included (Figure 1).

#### 2.1.2 Study variables

The dietary fatty acids of interest in this study include SFA, MUFA, PUFA, n-3 PUFA, and n-6 PUFA. Each participant underwent two 24-h dietary recall interviews: the first was conducted in person at the Mobile Examination Center, and the second over the phone 3–10 days later. In order to maintain precision, the analysis utilized the average of the two dietary data, with “dietary two-day sample weight” serving as weight.

Gallstone cases were identified using the inquiry “Has DR ever said you have gallstones”. Participants who responded affirmatively were classified as having gallstones.

#### 2.1.3 Covariates

To address potential confounding variables, the study incorporated the following covariates. These covariates encompassed continuous variables such as age, body mass index (BMI), triglyceride, high-density lipoprotein-cholesterol (HDL-C), dietary energy intake, fat intake, and cholesterol intake. Additionally, categorical variables such as gender, race, education level, marital status, poverty income ratio (PIR), hypertension, diabetes, hepatic steatosis, smoking, and drinking were also considered. Detailed definitions of covariates are available in Supplementary Table S1.

#### 2.1.4 Statistical analysis

The sample weights from the NHANES stratified multistage sampling were taken into account in the baseline characteristics and correlation analysis. The baseline characteristics of the participants were determined depending on their gallstone status. As the continuous variables were all skewed, median and interquartile range (IQR) were used to describe them, and frequency and weighted percentage were used to describe the categorical variables. Group comparisons were carried out employing the weighted χ^2^ test and the Wilcoxon rank-sum test.

Fully adjusted for all covariates, a weighted multivariate logistic regression model was used for analysis. We first analyzed fatty acid intake as a continuous variable for its association with gallstones. Subsequently, fatty acid intake was divided into quartiles to conduct a trend test. Given that gallstones are more prevalent in women and that there are gender disparities in fatty acid intake (11, 12), subgroup logistic regression analysis was conducted by gender. Additionally, restricted cubic splines (RCS) were employed to examine the potential nonlinear relationship in different genders.

Statistical analysis was conducted using R software (version 4.4.0) with the R packages “survey” (version 4.4-2) and “rms” (version 6.8-1). *p* values below 0.05 were deemed significant.

### 2.2 Mendelian randomization study

#### 2.2.1 Data sources

The fatty acid genome-wide association studies (GWAS) data were acquired from the IEU OpenGWAS project, which included a total of 115,006 European participants. The gallstone GWAS data were acquired from the Finngen R10 Release (13), comprising 40,191 European cases and 361,641 European controls overall. For these original GWAS studies, corresponding ethical approvals have been obtained. The population samples of exposure and outcome were from different consortia, ensuring minimal overlap. Detailed GWAS data information is presented in Table 1.

**Table 1:** GWAS data information in Mendelian randomization study.

Trait	GWAS ID	Consortium	Sample size	PMID	Ancestry
Gallstone	NA	Finngen	401,832 (40,191 cases)	36653562	European
Saturated fatty acid	ebi-a-GCST90092980	NA	115,006	35213538	European
Monounsaturated fatty acid	ebi-a-GCST90092928	NA	115,006	35213538	European
Polyunsaturated fatty acid	ebi-a-GCST90092939	NA	115,006	35213538	European
n-3 Polyunsaturated fatty acid	ebi-a-GCST90092931	NA	115,006	35213538	European
n-6 Polyunsaturated fatty acid	ebi-a-GCST90092933	NA	115,006	35213538	European
Total fatty acid	ebi-a-GCST90092987	NA	115,006	35213538	European

#### 2.2.2 Instrumental variables selection

Single nucleotide polymorphisms (SNPs) strongly associated with the exposure factors were employed as unconfounded instrumental variables (IVs) for the analysis. The MR analysis was based on three fundamental assumptions: (1) IVs are substantially linked with exposure; (2) IVs do not influence outcomes via confounders; (3) IVs impact outcomes only through their effect on exposure. Based on the above criteria, the candidate SNP must reach the genome-wide significance (5 × 10^−8^). To guarantee the independence of candidate SNPs, the linkage disequilibrium threshold was set to be r^2^ = 0.001 and clumping distance = 10,000 kb. The intensity of each SNP was calculated using the formula: F statistic = Beta^2^/SE^2^ (14), and SNPs with *F* < 10 were discarded as weak IVs. To ensure adherence to the core assumptions of MR, we screened for and removed confounders using the GWAS Catalog (15). Detailed information on the confounding SNPs and traits is provided in Supplementary Table S2.

#### 2.2.3 Mendelian randomization analysis

The main method for MR analysis was the inverse variance weighted (IVW) approach. It combines Wald ratios of each IV to conduct a meta-analysis. It is considered the most accurate statistical method when horizontal pleiotropy does not exist (16). Additionally, supplementary analytical methods such as weighted median, MR-Egger, and MR Robust Adjusted Profile Score (MR-RAPS) were also employed in this study (17–19). We employed MR-PRESSO and RadialMR to detect and eliminate outliers (20, 21). Horizontal pleiotropy was evaluated using the MR Egger intercept and MR-PRESSO global test, while heterogeneity was assessed with Cochran’s Q test (22). *p* values below 0.05 suggest the existence of pleiotropy or heterogeneity. The impact of individual outlier IVs was assessed using the funnel plot and the leave-one-out analysis. We applied STROBE-MR to design this study (Supplementary Table S3) (23). The R packages used for MR analysis were “TwoSampleMR” (version 0.6.3), “mr. raps” (version 0.2), “MRPRESSO” (version 1.0), and “RadialMR” (version 1.1).

## 3 Results

### 3.1 Cross-sectional study

#### 3.1.1 Baseline characteristics

Table 2 presents the weighted baseline characteristics of the study participants grouped by gallstone status. The study involved 6,629 participants, comprising 5,894 individuals without gallstones and 735 individuals with gallstones. The analysis showed that individuals with gallstones were more likely to be older, female, obese, former smokers, and former drinkers, and exhibited a higher prevalence of hypertension, diabetes, and hepatic steatosis compared to those without gallstones (*p* < 0.001). Additionally, the two groups differed significantly in race, education level, marital status, and PIR. The intake of energy, fat, cholesterol, MUFA, and n-6 PUFA was found to be lower in the gallstone group.

**Table 2:** Weighted basic characteristics of participants by gallstone.

Characteristic	Overall (*n* = 6,629)	Non-gallstone (*n* = 5,894)	Gallstone (*n* = 735)	*p*-value
Age (years), Median (IQR)	48.00 (33.00, 63.00)	47.00 (32.00, 61.00)	60.00 (47.00, 70.00)	<0.001
Gender, *n* (weighted %)				<0.001
Male	3,166.00 (47.90%)	2,954.00 (50.40%)	212.00 (27.60%)	
Female	3,463.00 (52.10%)	2,940.00 (49.60%)	523.00 (72.40%)	
Race, *n* (weighted %)				0.001
Mexican American	736.00 (8.04%)	652.00 (8.10%)	84.00 (7.49%)	
Other Hispanic	656.00 (7.74%)	574.00 (7.84%)	82.00 (6.95%)	
Non-Hispanic White	2,390.00 (62.92%)	2,064.00 (62.22%)	326.00 (68.58%)	
Non-Hispanic Black	1,864.00 (11.50%)	1,706.00 (11.91%)	158.00 (8.20%)	
Non-Hispanic Asian	666.00 (5.82%)	629.00 (6.30%)	37.00 (1.97%)	
Other Race	317.00 (3.98%)	269.00 (3.63%)	48.00 (6.81%)	
Education level, *n* (weighted %)				0.012
Below high school	1,076.00 (9.40%)	962.00 (9.52%)	114.00 (8.41%)	
High school	1,564.00 (26.67%)	1,379.00 (25.80%)	185.00 (33.71%)	
Above high school	3,982.00 (63.93%)	3,546.00 (64.67%)	436.00 (57.88%)	
Marital status, *n* (weighted %)				<0.001
Married or living with a partner	3,842.00 (62.59%)	3,418.00 (62.81%)	424.00 (60.83%)	
Widowed/Divorced/Separated	1,501.00 (17.89%)	1,285.00 (16.92%)	216.00 (25.74%)	
Never married	1,281.00 (19.51%)	1,186.00 (20.27%)	95.00 (13.43%)	
PIR, *n* (weighted %)				<0.001
<1.3	1,609.00 (18.76%)	1,431.00 (18.46%)	178.00 (21.12%)	
1.3–3.5	2,288.00 (34.09%)	2,001.00 (33.12%)	287.00 (41.69%)	
>3.5	1,952.00 (47.15%)	1,756.00 (48.41%)	196.00 (37.19%)	
BMI (kg/m^2^), Median (IQR)	28.70 (24.70, 33.40)	28.40 (24.40, 32.90)	31.20 (27.00, 37.72)	<0.001
Triglyceride (mg/dl), Median (IQR)	90.00 (61.00, 139.00)	90.00 (60.00, 136.00)	97.00 (67.00, 152.56)	0.110
HDL-C (mg/dl), Median (IQR)	51.00 (42.00, 63.00)	51.00 (42.00, 63.00)	50.00 (43.00, 62.00)	0.300
Hypertension, n (weighted %)				<0.001
No	2,816.00 (50.48%)	2,590.00 (52.32%)	226.00 (35.58%)	
Yes	3,812.00 (49.52%)	3,303.00 (47.68%)	509.00 (64.42%)	
Diabetes, *n* (weighted %)				<0.001
No	5,262.00 (84.56%)	4,773.00 (85.98%)	489.00 (73.01%)	
Yes	1,367.00 (15.44%)	1,121.00 (14.02%)	246.00 (26.99%)	
Hepatic steatosis, *n* (weighted %)				<0.001
No	3,285.00 (56.80%)	3,022.00 (58.39%)	263.00 (43.81%)	
Yes	2,592.00 (43.20%)	2,224.00 (41.61%)	368.00 (56.19%)	
Smoking, *n* (weighted %)				<0.001
Never	3,831.00 (58.32%)	3,446.00 (59.02%)	385.00 (52.70%)	
Former	1,612.00 (25.46%)	1,379.00 (24.63%)	233.00 (32.20%)	
Current	1,182.00 (16.21%)	1,067.00 (16.35%)	115.00 (15.10%)	
Drinking, *n* (weighted %)				<0.001
Never	561.00 (6.90%)	500.00 (6.74%)	61.00 (8.20%)	
Former	1,281.00 (15.56%)	1,079.00 (13.94%)	202.00 (28.63%)	
Mild	2,411.00 (39.20%)	2,172.00 (39.84%)	239.00 (34.04%)	
Moderate	1,121.00 (19.38%)	1,003.00 (19.64%)	118.00 (17.26%)	
Heavy	1,081.00 (18.96%)	990.00 (19.84%)	91.00 (11.87%)	
Energy (kcal/day), Median (IQR)	1,940.50 (1,500.62, 2,501.00)	1,950.77 (1,506.39, 2,515.99)	1,811.58 (1,407.37, 2,332.72)	<0.001
Total fat (g/day), Median (IQR)	78.49 (58.43, 103.59)	79.12 (59.02, 105.53)	75.52 (55.38, 98.22)	0.022
Cholesterol (mg/day), Median (IQR)	262.00 (170.00, 407.00)	268.00 (173.00, 413.02)	221.61 (141.81, 341.71)	0.003
SFA (g/day), Median (IQR)	25.00 (17.79, 34.58)	25.09 (17.84, 34.72)	23.83 (17.50, 32.39)	0.200
MUFA (g/day), Median (IQR)	26.58 (19.34, 36.28)	26.76 (19.46, 36.66)	25.44 (17.36, 33.83)	0.006
PUFA (g/day), Median (IQR)	18.11 (12.53, 24.69)	18.24 (12.63, 24.89)	16.92 (11.93, 23.18)	0.028
n-3 PUFA (g/day), Median (IQR)	1.73 (1.14, 2.47)	1.75 (1.16, 2.49)	1.62 (1.07, 2.40)	0.088
n-6 PUFA (g/day), Median (IQR)	16.23 (11.12, 22.11)	16.33 (11.20, 22.25)	15.48 (10.70, 20.50)	0.024
Total fatty acid (g/day), Median (IQR)	70.86 (52.48, 93.86)	71.17 (52.95, 95.36)	67.04 (49.40, 87.36)	0.026

IQR, interquartile range; PIR, poverty income ratio; BMI, body mass index; HDL-C, high-density lipoprotein-cholesterol; SFA, saturated fatty acid; MUFA, monounsaturated fatty acid; PUFA, polyunsaturated fatty acid. Sample counts (n) are unweighted. Percentages, medians (IQR), and *p*-values are based on weighted data. Wilcoxon rank-sum test for complex survey samples; chi-squared test with Rao & Scott’s second-order correction.

#### 3.1.2 Association between dietary fatty acids and gallstone

**Figure 2:**
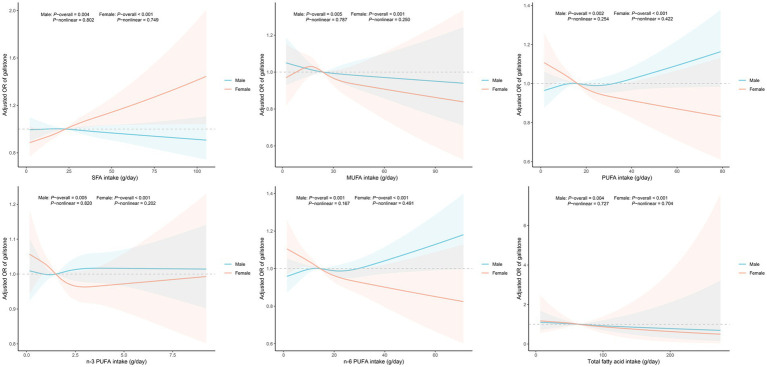
Dose-response analysis of fatty acids intake and gallstone risk. Models grouped by gender were adjusted for age, race, education level, marital status, poverty income ratio, body mass index, triglyceride, high-density lipoprotein-cholesterol, hypertension, diabetes, hepatic steatosis, smoking, drinking, energy intake, total fat intake and cholesterol intake. Abbreviation: OR, odds ratio; CI, confidence interval; SFA, saturated fatty acid; MUFA, monounsaturated fatty acid; PUFA, polyunsaturated fatty acid.

After adjusting for all covariates, the results of the weighted logistic regression analysis are presented in Table 3. The results showed that in the female subgroup, higher consumption of SFA was positively associated with an increased risk of gallstones (OR = 1.054, 95% CI: 1.003–1.107), while higher n-3 PUFA (OR = 0.647, 95% CI: 0.420–0.996) and n-6 PUFA (OR = 0.926, 95% CI: 0.871–0.984) intake was linked to a reduced risk. When analyzing fatty acid intake by quartiles, compared to Q1, Q3 and Q4 groups of n-3 PUFA and Q3 group of n-6 PUFA were inversely associated with gallstone risk among females. However, no notable associations were found in the male subgroup. Figure 2 displays the RCS study illustrating the impact of dietary fatty acids on gallstone risk according to gender. After accounting for all confounding variables, no nonlinear correlations were discovered in any of the groups (*P*-nonlinear >0.05).

**Table 3:** Weighted logistic regression analysis of the association between fatty acid intake and gallstone.

Fatty acids	Continuous	*p*-value	Q1	Q2	Q3	Q4	*p* for trend
(g/day)	OR (95% CI)			OR (95% CI)	OR (95% CI)	OR (95% CI)	
SFA	1.032 (0.979, 1.088)	0.204	Ref	1.134 (0.424, 3.034)	1.145 (0.411, 3.190)	1.396 (0.381, 5.117)	0.642
Male	0.994 (0.906, 1.091)	0.889	Ref	2.593 (0.497, 13.530)	0.896 (0.086, 9.333)	1.703 (0.190, 15.251)	0.878
Female	**1.054 (1.003, 1.107)**	**0.040**	Ref	1.014 (0.378, 2.716)	1.441 (0.432, 4.804)	1.584 (0.394, 6.366)	0.405
MUFA	0.980 (0.914, 1.051)	0.525	Ref	0.592 (0.207, 1.691)	0.859 (0.300, 2.459)	0.558 (0.093, 3.355)	0.664
Male	0.963 (0.850, 1.091)	0.508	Ref	0.989 (0.148, 6.636)	0.854 (0.119, 6.121)	0.444 (0.060, 3.309)	0.295
Female	0.998 (0.940, 1.060)	0.947	Ref	0.572 (0.174, 1.882)	1.104 (0.304, 4.008)	0.876 (0.098, 7.814)	0.853
PUFA	0.960 (0.908, 1.016)	0.134	Ref	0.539 (0.208, 1.393)	**0.346 (0.132, 0.912)** ^**a**^	**0.217 (0.049, 0.966)** ^**b**^	**0.027**
Male	1.008 (0.933, 1.089)	0.811	Ref	0.490 (0.112, 2.138)	0.967 (0.215, 4.357)	0.252 (0.042, 1.518)	0.210
Female	**0.928 (0.878, 0.981)**	**0.015**	Ref	0.522 (0.195, 1.397)	**0.216 (0.082, 0.565)**^**c**^	0.208 (0.039, 1.109)	**0.019**
n-3 PUFA	0.755 (0.501, 1.136)	0.148	Ref	0.634 (0.257, 1.564)	0.501 (0.248, 1.011)	0.408 (0.135, 1.229)	0.070
Male	0.856 (0.513, 1.430)	0.504	Ref	0.269 (0.039, 1.833)	0.623 (0.113, 3.435)	0.416 (0.058, 3.001)	0.571
Female	**0.647 (0.420, 0.996)**	**0.048**	Ref	0.699 (0.232, 2.103)	**0.419 (0.216, 0.815)**^**d**^	**0.335 (0.122, 0.919)**^**e**^	**0.014**
n-6 PUFA	0.960 (0.902, 1.022)	0.167	Ref	0.538 (0.188, 1.543)	0.443 (0.174, 1.126)	0.313 (0.077, 1.269)	0.066
Male	1.016 (0.928, 1.111)	0.703	Ref	0.523 (0.119, 2.294)	0.966 (0.213, 4.369)	0.276 (0.047, 1.636)	0.250
Female	**0.926 (0.871, 0.984)**	**0.019**	Ref	0.546 (0.187, 1.590)	**0.319 (0.125, 0.817)**^**f**^	0.354 (0.078, 1.612)	0.064
Total fatty acid	0.908 (0.775, 1.063)	0.191	Ref	0.888 (0.267, 2.954)	0.779 (0.205, 2.958)	0.593 (0.057, 6.175)	0.598
Male	0.850 (0.696, 1.038)	0.097	Ref	2.059 (0.284, 14.916)	0.784 (0.086, 7.177)	1.231 (0.059, 25.457)	0.747
Female	0.951 (0.772, 1.171)	0.593	Ref	0.744 (0.200, 2.763)	0.837 (0.164, 4.262)	0.475 (0.024, 9.459)	0.753

Q1 to Q4, quintile 1 to 4; OR, odds ratio; CI, confidence interval; Ref, reference; SFA, saturated fatty acid; MUFA, monounsaturated fatty acid; PUFA, polyunsaturated fatty acid. Models grouped by gender were adjusted for age, race, education level, marital status, poverty income ratio, body mass index, triglyceride, high-density lipoprotein-cholesterol, hypertension, diabetes, hepatic steatosis, smoking, drinking, energy intake, total fat intake and cholesterol intake. The overall group was additionally adjusted for gender. Bold values indicate statistical significance.

a *p* value: 0.037.

b *p* value: 0.047.

c *p* value: 0.008.

d *p* value: 0.019.

e *p* value: 0.038.

f *p* value: 0.025.

### 3.2 Mendelian randomization study

After applying the selection criteria for IVs and removing outliers, the SNPs utilized in the MR analysis are listed in Supplementary Tables S4–S9. All SNPs exhibited F-statistics over 10, indicating no weak IVs.

**Figure 3:**
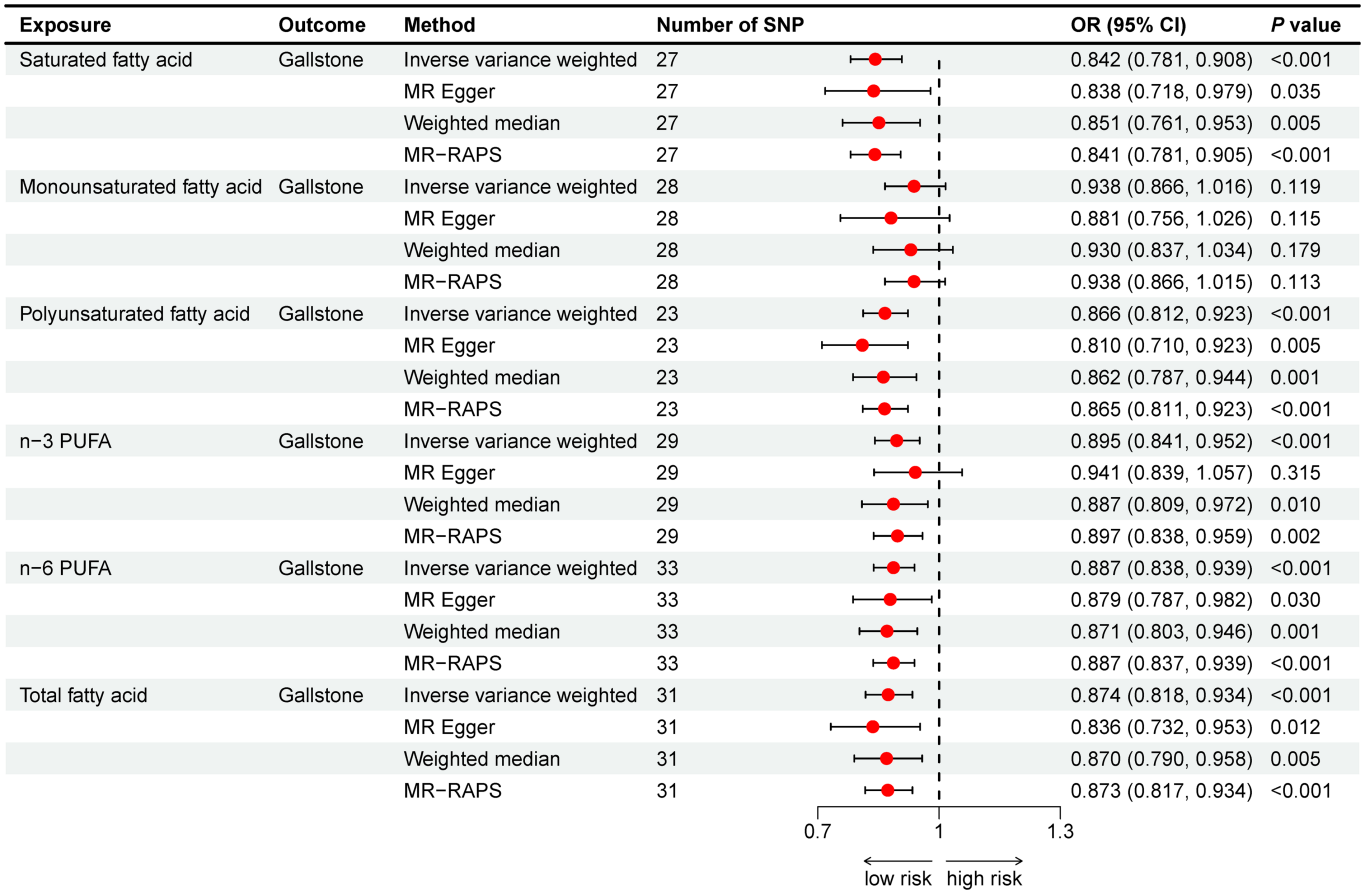
Mendelian randomization analysis of fatty acids and gallstone risk. Abbreviation: OR, odds ratio; CI, confidence interval; PUFA, polyunsaturated fatty acid; MR-RAPS, MR Robust adjusted profile score.

The IVW method showed that SFA (OR = 0.842, 95% CI: 0.781–0.908), n-3 PUFA (OR = 0.895, 95% CI: 0.841–0.952), and n-6 PUFA (OR = 0.887, 95% CI: 0.838–0.939) could reduce gallstone risk (all *p* < 0.001), as shown in Figure 3. All three supplementary MR methods demonstrated results consistent with the IVW method, enhancing the reliability of the findings. Table 4 shows that there is no indication of pleiotropy or heterogeneity. No potential outliers were observed that could affect the results, as shown by the leave-one-out results and funnel plots (Supplementary Figures S1, S2).

**Table 4:** Assessment of pleiotropy and heterogeneity in Mendelian randomization study.

Exposure	Outcome	Horizontal pleiotropy		Heterogeneity (Cochrane’s Q)			
		MR Egger	MR-PRESSO	MR Egger		Inverse variance weighted	
		*p* value	*p* value	Q	*p* value	Q	*p* value
Saturated fatty acid	Gallstone	0.949	0.396	27.969	0.309	27.973	0.360
Monounsaturated fatty acid	Gallstone	0.347	0.345	28.882	0.316	29.902	0.319
Polyunsaturated fatty acid	Gallstone	0.268	0.930	12.150	0.935	13.446	0.920
n-3 PUFA	Gallstone	0.316	0.462	26.703	0.480	27.749	0.478
n-6 PUFA	Gallstone	0.855	0.659	28.058	0.618	28.092	0.665
Total fatty acid	Gallstone	0.444	0.720	24.675	0.695	25.278	0.711

## 4 Discussion

This study is the first to utilize NHANES large sample data and the latest GWAS data to investigate the relationship between various dietary fatty acids and gallstones in the American population, and to explore the causal relationship without confounding factors using MR analysis. Results from the NHANES study indicated that for females, higher SFA intake was linked to higher gallstone risk, whereas higher n-3 and n-6 PUFA intake were linked to a reduced risk. No notable associations were found in the male group. MR analysis indicated that SFA, n-3 and n-6 PUFA could reduce gallstone risk.

Studies have demonstrated that the main causes contributing to the development of gallstones are supersaturation of bile components, promotion of crystal nucleation by mucin and similar substances, and reduced gallbladder motility (24). Multiple epidemiological studies have confirmed that PUFA can prevent gallstone formation (10, 25, 26). This may be attributed to the following mechanisms: firstly, the intake of fish oil, which contains high levels of n-3 PUFA, can lower cholesterol saturation in bile, inhibiting the formation of cholesterol crystals (8, 27). Secondly, n-3 PUFA can also increase the secretion of bile acids and phospholipids, inhibit the formation of biliary mucin, and improve gallbladder motility to prevent gallstone formation (9, 28, 29). Thirdly, PUFA can reduce serum and liver cholesterol and triglyceride levels, thereby lowering the risk of gallstones (9, 30–32). Additionally, since inflammation plays a significant role in gallstone formation (33, 34), PUFA may also prevent gallstones through their anti-inflammatory effects (35, 36).

Regarding SFA, two clinical studies conducted in France and Italy found a positive correlation between dietary SFA and gallstones (37, 38). An American clinical investigation found that consuming more long-chain SFA is linked to a greater likelihood of developing gallstones in men, whereas intake of medium- or short-chain SFA does not seem to affect this risk (39). The close correlation between SFA and metabolic syndrome has been highlighted (40). SFA has been found to be linked to several disorders connected to metabolic syndrome, such as cardiovascular disease (41), insulin resistance (42, 43), and cancer (44). Since several components of metabolic syndrome are risk factors for gallstones, gallstones can be considered a biliary manifestation of metabolic syndrome (45, 46). Anyway, the precise impact of MUFA remains uncertain, with some clinical studies finding a negative correlation between MUFA and gallstone risk (10, 38), while others have found the opposite result (47, 48).

Our study findings suggest that in females, consuming dietary PUFA can lower the likelihood of developing gallstones, while higher intake of SFA is associated with an increased risk. These findings align with prior research. However, no such correlation was observed in males. Differences in estrogen and lipid metabolism may lead to a higher prevalence of gallstones in females (49, 50), and this disparity may influence the effects of fatty acids on different genders. Moreover, the cross-sectional study and the MR study yielded contradictory findings regarding the role of SFA. This discrepancy can be attributed to the inherent limitations of each study design. Despite adjusting for potential covariates as thoroughly as possible, the cross-sectional study remains susceptible to residual confounding. In contrast, the MR study indicates a causal relationship between lifetime exposure and outcome with minimal confounding, but it cannot account for gender differences and cannot fully eliminate the influence of pleiotropy. However, further clinical and experimental studies are required to validate these gender differences and explore possible mechanisms.

This study utilized a nationally representative NHANES sample for weighted analysis and conducted subgroup analysis by gender, making the results generalizable and instructive. Combining observational studies with MR analysis enhanced the reliability of the findings. However, there are some limitations to this study that should not be overlooked. Firstly, the dietary and gallstone data were self-reported, which introduces the possibility of recall bias and lack of precise imaging diagnosis. Secondly, the 24-h dietary recall interview data may not accurately reflect an individual’s long-term dietary habits. Thirdly, this study only targeted adults in the United States, so caution is needed when generalizing the results to other populations.

## 5 Conclusion

Our study found that in females, dietary SFA was positively associated with gallstone risk, while higher intake of n-3 and n-6 PUFA was associated with a decreased risk. No significant associations were found in men. MR analysis supports that SFA, n-3 and n-6 PUFA could reduce gallstone risk. These findings provide new perspectives on dietary strategies for the prevention and treatment of gallstones. However, further prospective cohort studies and experimental research are required to validate these results and explore the underlying mechanisms.

## Data Availability

The original contributions presented in the study are included in the article/Supplementary material, further inquiries can be directed to the corresponding author.
