# Supplementary material for: Dietary fatty acids and gallstone risk: insights from NHANES and Mendelian randomization analysis
Source: Front Nutr. 2024 Aug 15;11:1454648. doi: 10.3389/fnut.2024.1454648 (PMC11358065; doi:10.3389/fnut.2024.1454648)
Supplement: Supplementary file 1 [file Data_Sheet_1.docx]

**Supplementary Table S3**

**STROBE-MR checklist of recommended items to address in reports of Mendelian randomization studies**^1^ ^2^

| **Item No.** | **Section** | **Checklist item** | **Relevant text from manuscript** |
| --- | --- | --- | --- |
| 1 | **TITLE and ABSTRACT** | Indicate Mendelian randomization (MR) as the study’s design in the title and/or the abstract if that is a main purpose of the study | Dietary fatty acids and gallstone risk: insights from NHANES and Mendelian randomization analysis  This study aims to explore the associations between fatty acids and gallstones using a large sample of American population and Mendelian randomization (MR) methods. |
|  | **INTRODUCTION** |  |  |
| 2 | **Background** | Explain the scientific background and rationale for the reported study. What is the exposure? Is a potential causal relationship between exposure and outcome plausible? Justify why MR is a helpful method to address the study question | A growing emphasis is placed on the influence of dietary fatty acids on gallstone formation. Prior research has explored the potential protective role of polyunsaturated fatty acids (PUFA) in preventing gallstones [7–10]. However, the evidence on saturated fatty acids (SFA) and monounsaturated fatty acids (MUFA) is limited by small sample sizes and the influence of confounding factors.  Mendelian randomization (MR) was then applied to investigate the causality of fatty acids on gallstones, adjusting for residual confounding factors and addressing the limitations of the cross-sectional study. |
| 3 | **Objectives** | State specific objectives clearly, including pre-specified causal hypotheses (if any). State that MR is a method that, under specific assumptions, intends to estimate causal effects | Mendelian randomization (MR) was then applied to investigate the causality of fatty acids on gallstones, adjusting for residual confounding factors and addressing the limitations of the cross-sectional study. |
|  | **METHODS** |  |  |
| 4 | **Study design and data sources** | Present key elements of the study design early in the article. Consider including a table listing sources of data for all phases of the study. For each data source contributing to the analysis, describe the following: |  |
|  | a) | Setting: Describe the study design and the underlying population, if possible. Describe the setting, locations, and relevant dates, including periods of recruitment, exposure, follow-up, and data collection, when available. | Detailed GWAS data information is presented in Table 1. |
|  | b) | Participants: Give the eligibility criteria, and the sources and methods of selection of participants. Report the sample size, and whether any power or sample size calculations were carried out prior to the main analysis | The fatty acid genome-wide association studies (GWAS) data were acquired from the IEU OpenGWAS project, which included a total of 115,006 European participants. The gallstone GWAS data were acquired from the Finngen R10 Release [13], comprising 361,641 European controls and 40,191 European cases overall. |
|  | c) | Describe measurement, quality control and selection of genetic variants | Based on the above criteria, the candidate SNP must reach the genome-wide significance (5×10-8). To guarantee the independence of candidate SNPs, the linkage disequilibrium threshold was set to be r^2^ = 0.001 and clumping distance = 10,000 kb. The intensity of each SNP was calculated using the formula: F statistic = Beta^2^/SE^2^[14], and SNPs with F < 10 were discarded as weak IVs. To ensure adherence to the core assumptions of MR, we screened for and removed confounders using the GWAS Catalog[15]. Detailed information on the confounding SNPs and traits is provided in Supplementary Table S2. |
|  | d) | For each exposure, outcome, and other relevant variables, describe methods of assessment and diagnostic criteria for diseases | Detailed GWAS data information is presented in Table 1. |
|  | e) | Provide details of ethics committee approval and participant informed consent, if relevant | For these original GWAS studies, corresponding ethical approvals have been obtained. |
| 5 | **Assumptions** | Explicitly state the three core IV assumptions for the main analysis (relevance, independence and exclusion restriction) as well assumptions for any additional or sensitivity analysis | The MR analysis was based on three fundamental assumptions: (1) IVs are substantially linked with exposure; (2) IVs do not influence outcomes via confounders; (3) IVs impact outcomes only through their effect on exposure. |
| 6 | **Statistical methods: main analysis** | Describe statistical methods and statistics used |  |
|  | a) | Describe how quantitative variables were handled in the analyses (i.e., scale, units, model) | NA |
|  | b) | Describe how genetic variants were handled in the analyses and, if applicable, how their weights were selected | Based on the above criteria, the candidate SNP must reach the genome-wide significance (5×10-8). To guarantee the independence of candidate SNPs, the linkage disequilibrium threshold was set to be r^2^ = 0.001 and clumping distance = 10,000 kb. The intensity of each SNP was calculated using the formula: F statistic = Beta^2^/SE^2^[14], and SNPs with F < 10 were discarded as weak IVs. To ensure adherence to the core assumptions of MR, we screened for and removed confounders using the GWAS Catalog[15]. Detailed information on the confounding SNPs and traits is provided in Supplementary Table S2. |
|  | c) | Describe the MR estimator (e.g. two-stage least squares, Wald ratio) and related statistics. Detail the included covariates and, in case of two-sample MR, whether the same covariate set was used for adjustment in the two samples | The main method for MR analysis was the inverse variance weighted (IVW) approach. It combines Wald ratios of each IV to conduct a meta-analysis. It is considered the most accurate statistical method when horizontal pleiotropy does not exist [16]. |
|  | d) | Explain how missing data were addressed | NA |
|  | e) | If applicable, indicate how multiple testing was addressed | NA |
| 7 | **Assessment of assumptions** | Describe any methods or prior knowledge used to assess the assumptions or justify their validity | The main method for MR analysis was the inverse variance weighted (IVW) approach. It combines Wald ratios of each IV to conduct a meta-analysis. It is considered the most accurate statistical method when horizontal pleiotropy does not exist [16]. Additionally, supplementary analytical methods such as weighted median, MR-Egger, and MR Robust Adjusted Profile Score (MR-RAPS) were also employed in this study [17–19]. |
| 8 | **Sensitivity analyses and additional analyses** | Describe any sensitivity analyses or additional analyses performed (e.g. comparison of effect estimates from different approaches, independent replication, bias analytic techniques, validation of instruments, simulations) | We employed MR-PRESSO and RadialMR to detect and eliminate outliers [20,21]. Horizontal pleiotropy was evaluated using the MR Egger intercept and MR-PRESSO global test, while heterogeneity was assessed with Cochran's Q test [22]. P values below 0.05 suggest the existence of pleiotropy or heterogeneity. The impact of individual outlier IVs was assessed using the funnel plot and the leave-one-out analysis. |
| 9 | **Software and pre-registration** |  |  |
|  | a) | Name statistical software and package(s), including version and settings used | Statistical analysis was conducted using R software (version 4.4.0). The R packages used for MR analysis were "TwoSampleMR" (version 0.6.3), "mr.raps" (version 0.2), "MRPRESSO" (version 1.0), and "RadialMR" (version 1.1). |
|  | b) | State whether the study protocol and details were pre-registered (as well as when and where) | NA |
|  | **RESULTS** |  |  |
| 10 | **Descriptive data** |  |  |
|  | a) | Report the numbers of individuals at each stage of included studies and reasons for exclusion. Consider use of a flow diagram | The fatty acid genome-wide association studies (GWAS) data were acquired from the IEU OpenGWAS project, which included a total of 115,006 European participants. The gallstone GWAS data were acquired from the Finngen R10 Release [13], comprising 361,641 European controls and 40,191 European cases overall. |
|  | b) | Report summary statistics for phenotypic exposure(s), outcome(s), and other relevant variables (e.g. means, SDs, proportions) | NA |
|  | c) | If the data sources include meta-analyses of previous studies, provide the assessments of heterogeneity across these studies | The data sources do not include meta-analyses of previous studies. |
|  | d) | For two-sample MR:  i.  Provide justification of the similarity of the genetic variant-exposure associations between the exposure and outcome samples  ii.  Provide information on the number of individuals who overlap between the exposure and outcome studies | The population samples of exposure and outcome were from different consortia, ensuring minimal overlap. |
| 11 | **Main results** |  |  |
|  | a) | Report the associations between genetic variant and exposure, and between genetic variant and outcome, preferably on an interpretable scale | The IVW method showed that SFA (OR = 0.842, 95% CI: 0.781-0.908), n-3 PUFA (OR = 0.895, 95% CI: 0.841-0.952), and n-6 PUFA (OR = 0.887, 95% CI: 0.838-0.939) could reduce gallstone risk (all P < 0.001), as shown in Figure 3. All three supplementary MR methods demonstrated results consistent with the IVW method, enhancing the reliability of the findings. |
|  | b) | Report MR estimates of the relationship between exposure and outcome, and the measures of uncertainty from the MR analysis, on an interpretable scale, such as odds ratio or relative risk per SD difference | The IVW method showed that SFA (OR = 0.842, 95% CI: 0.781-0.908), n-3 PUFA (OR = 0.895, 95% CI: 0.841-0.952), and n-6 PUFA (OR = 0.887, 95% CI: 0.838-0.939) could reduce gallstone risk (all P < 0.001), as shown in Figure 3. All three supplementary MR methods demonstrated results consistent with the IVW method, enhancing the reliability of the findings. |
|  | c) | If relevant, consider translating estimates of relative risk into absolute risk for a meaningful time period | NA |
|  | d) | Consider plots to visualize results (e.g. forest plot, scatterplot of associations between genetic variants and outcome versus between genetic variants and exposure) | Figure 3, Table 4, Supplementary Figure S1-S2. |
| 12 | **Assessment of assumptions** |  |  |
|  | a) | Report the assessment of the validity of the assumptions | Single nucleotide polymorphisms (SNPs) strongly associated with the exposure factors were employed as unconfounded instrumental variables (IVs) for the analysis.  After applying the selection criteria for IVs and removing outliers, the SNPs utilized in the MR analysis are listed in Supplementary Tables S2-S7. All SNPs exhibited F-statistics over 10, indicating no weak IVs. |
|  | b) | Report any additional statistics (e.g., assessments of heterogeneity across genetic variants, such as *I^2^*, Q statistic or E-value) | Table 4 shows that there is no indication of pleiotropy or heterogeneity. |
| 13 | **Sensitivity analyses and additional analyses** |  |  |
|  | a) | Report any sensitivity analyses to assess the robustness of the main results to violations of the assumptions | Additionally, supplementary analytical methods such as weighted median, MR-Egger, and MR Robust Adjusted Profile Score (MR-RAPS) were also employed in this study[17–19]. We employed MR-PRESSO and RadialMR to detect and eliminate outliers [20,21]. Horizontal pleiotropy was evaluated using the MR Egger intercept and MR-PRESSO global test, while heterogeneity was assessed with Cochran's Q test [22]. P values below 0.05 suggest the existence of pleiotropy or heterogeneity. The impact of individual outlier IVs was assessed using the funnel plot and the leave-one-out analysis. |
|  | b) | Report results from other sensitivity analyses or additional analyses | All three supplementary MR methods demonstrated results consistent with the IVW method, enhancing the reliability of the findings. Table 4 shows that there is no indication of pleiotropy or heterogeneity. No potential outliers were observed that could affect the results, as shown by the leave-one-out results and funnel plots (Supplementary Figure S1-S2). |
|  | c) | Report any assessment of direction of causal relationship (e.g., bidirectional MR) | NA |
|  | d) | When relevant, report and compare with estimates from non-MR analyses | After adjusting for all covariates, the results of the weighted logistic regression analysis are presented in Table 3. The results showed that in the female subgroup, higher consumption of SFA was positively associated with an increased risk of gallstones (OR = 1.054, 95% CI: 1.003-1.107), while higher n-3 PUFA (OR = 0.647, 95% CI: 0.420-0.996) and n-6 PUFA (OR = 0.926, 95% CI: 0.871-0.984) intake was linked to a reduced risk. When analyzing fatty acid intake by quartiles, compared to Q1, Q3 and Q4 groups of n-3 PUFA and Q3 group of n-6 PUFA were inversely associated with gallstone risk among females. However, no notable associations were found in the male subgroup. Figure 2 displays the RCS study illustrating the impact of dietary fatty acids on gallstone risk according to gender. After accounting for all confounding variables, no nonlinear correlations were discovered in any of the groups (P-nonlinear > 0.05).  The IVW method showed that SFA (OR = 0.842, 95% CI: 0.781-0.908), n-3 PUFA (OR = 0.895, 95% CI: 0.841-0.952), and n-6 PUFA (OR = 0.887, 95% CI: 0.838-0.939) could reduce gallstone risk (all P < 0.001), as shown in Figure 3. All three supplementary MR methods demonstrated results consistent with the IVW method, enhancing the reliability of the findings. Table 4 shows that there is no indication of pleiotropy or heterogeneity. No potential outliers were observed that could affect the results, as shown by the leave-one-out results and funnel plots (Supplementary Figure S1-S2). |
|  | e) | Consider additional plots to visualize results (e.g., leave-one-out analyses) | Supplementary Figure S2 |
|  | **DISCUSSION** |  |  |
| 14 | **Key results** | Summarize key results with reference to study objectives | This study is the first to utilize NHANES large sample data and the latest GWAS data to investigate the relationship between various dietary fatty acids and gallstones in the American population, and to explore the causal relationship without confounding factors using MR analysis. MR analysis indicated that SFA, n-3 and n-6 PUFA could reduce gallstone risk. |
| 15 | **Limitations** | Discuss limitations of the study, taking into account the validity of the IV assumptions, other sources of potential bias, and imprecision. Discuss both direction and magnitude of any potential bias and any efforts to address them | Moreover, the cross-sectional study and the MR study yielded contradictory findings regarding the role of SFA. This discrepancy can be attributed to the inherent limitations of each study design. Despite adjusting for potential covariates as thoroughly as possible, the cross-sectional study remains susceptible to residual confounding. In contrast, the MR study indicates a causal relationship between lifetime exposure and outcome with minimal confounding, but it cannot account for gender differences and cannot fully eliminate the influence of pleiotropy. However, further clinical and experimental studies are required to validate these gender differences and explore possible mechanisms. |
| 16 | **Interpretation** |  |  |
|  | a) | Meaning: Give a cautious overall interpretation of results in the context of their limitations and in comparison with other studies | These findings provide new perspectives on dietary strategies for the prevention and treatment of gallstones. However, further prospective cohort studies and experimental research are required to validate these results and explore the underlying mechanisms. |
|  | b) | Mechanism: Discuss underlying biological mechanisms that could drive a potential causal relationship between the investigated exposure and the outcome, and whether the gene-environment equivalence assumption is reasonable. Use causal language carefully, clarifying that IV estimates may provide causal effects only under certain assumptions | Multiple epidemiological studies have confirmed that PUFA can prevent gallstone formation[10,24,25]. This may be attributed to the following mechanisms: firstly, the intake of fish oil, which contains high levels of n-3 PUFA, can lower cholesterol saturation in bile, inhibiting the formation of cholesterol crystals[8,26]. Secondly, n-3 PUFA can also increase the secretion of bile acids and phospholipids, inhibit the formation of biliary mucin, and improve gallbladder motility to prevent gallstone formation[9,27,28]. Thirdly, PUFA can reduce serum and liver cholesterol and triglyceride levels, thereby lowering the risk of gallstones[9,29–31]. Additionally, since inflammation plays a significant role in gallstone formation[32,33], PUFA may also prevent gallstones through their anti-inflammatory effects[34,35].  The close correlation between SFA and metabolic syndrome has been highlighted[39]. SFA has been found to be linked to several disorders connected to metabolic syndrome, such as cardiovascular disease[40], insulin resistance[41,42], and cancer[43]. Since several components of metabolic syndrome are risk factors for gallstones, gallstones can be considered a biliary manifestation of metabolic syndrome[44,45]. |
|  | c) | Clinical relevance: Discuss whether the results have clinical or public policy relevance, and to what extent they inform effect sizes of possible interventions | Our study found that in females, dietary SFA was positively associated with gallstone risk, while higher intake of n-3 and n-6 PUFA was associated with a decreased risk. No significant associations were found in men. MR analysis supports that SFA, n-3 and n-6 PUFA could reduce gallstone risk. These findings provide new perspectives on dietary strategies for the prevention and treatment of gallstones. |
| 17 | **Generalizability** | Discuss the generalizability of the study results (a) to other populations, (b) across other exposure periods/timings, and (c) across other levels of exposure | Moreover, the cross-sectional study and the MR study yielded contradictory findings regarding the role of SFA. This discrepancy can be attributed to the inherent limitations of each study design. Despite adjusting for potential covariates as thoroughly as possible, the cross-sectional study remains susceptible to residual confounding. In contrast, the MR study indicates a causal relationship between lifetime exposure and outcome with minimal confounding, but it cannot account for gender differences and cannot fully eliminate the influence of pleiotropy. However, further clinical and experimental studies are required to validate these gender differences and explore possible mechanisms. |
|  | **OTHER INFORMATION** |  |  |
| 18 | **Funding** | Describe sources of funding and the role of funders in the present study and, if applicable, sources of funding for the databases and original study or studies on which the present study is based | There is no funding for this research. |
| 19 | **Data and data sharing** | Provide the data used to perform all analyses or report where and how the data can be accessed, and reference these sources in the article. Provide the statistical code needed to reproduce the results in the article, or report whether the code is publicly accessible and if so, where | The publicly available data used in this study can be accessed from NHANES (https://www.cdc.gov/nchs/nhanes), OpenGWAS (https://gwas.mrcieu.ac.uk/), and Finngen (https://www.finngen.fi/). |
| 20 | **Conflicts of Interest** | All authors should declare all potential conflicts of interest | The authors declare no conflict of interest. |

This checklist is copyrighted by the Equator Network under the Creative Commons Attribution 3.0 Unported (CC BY 3.0) license.

1. Skrivankova VW, Richmond RC, Woolf BAR, Yarmolinsky J, Davies NM, Swanson SA, et al. Strengthening the Reporting of Observational Studies in Epidemiology using Mendelian Randomization (STROBE-MR) Statement. JAMA. 2021;under review.

2. Skrivankova VW, Richmond RC, Woolf BAR, Davies NM, Swanson SA, VanderWeele TJ, et al. Strengthening the Reporting of Observational Studies in Epidemiology using Mendelian Randomisation (STROBE-MR): Explanation and Elaboration. BMJ. 2021;375:n2233.
